# Variation in foraging success among predators and its implications for population dynamics

**DOI:** 10.1002/ece3.2633

**Published:** 2016-12-20

**Authors:** Toshinori Okuyama

**Affiliations:** ^1^ Department of EntomologyNational Taiwan UniversityTaipeiTaiwan

**Keywords:** functional response, individual variation, individual‐based model, numerical response

## Abstract

The effects of the expected predation rate on population dynamics have been studied intensively, but little is known about the effects of predation rate variability (i.e., predator individuals having variable foraging success) on population dynamics. In this study, variation in foraging success among predators was quantified by observing the predation of the wolf spider *Pardosa pseudoannulata* on the cricket *Gryllus bimaculatus* in the laboratory. A population model was then developed, and the effect of foraging variability on predator–prey dynamics was examined by incorporating levels of variation comparable to those quantified in the experiment. The variability in the foraging success among spiders was greater than would be expected by chance (i.e., the random allocation of prey to predators). The foraging variation was density‐dependent; it became higher as the predator density increased. A population model that incorporates foraging variation shows that the variation influences population dynamics by affecting the numerical response of predators. In particular, the variation induces negative density‐dependent effects among predators and stabilizes predator–prey dynamics.

## 1 Introduction

Functional and numerical responses are important building blocks of population dynamics and are usually defined on a per capita basis. When *f* is a functional response that describes the predation rate per predator, the predation rate of a population of predators is obtained simply by *fP*, where *P* is the predator density (i.e., the functional response *f* is the predation rate of a predator population, when there is one predator per unit area, *P* = 1). Similarly, the numerical response β is typically defined as the reproduction rate per predator. Consequently, the reproductive output of a population of predators can be obtained as the product of the population density and the per capita effect, β*P*. These conventions are used in most theoretical investigations of consumer‐resource dynamics (Case, 2000; McCann, 2012; Murdoch, Briggs, & Nisbet, 2003).

Empirical studies closely follow these conventions and quantify functional and numerical responses by emphasizing the per capita concept. The predominant experimental approach is to place one predator and a variable number of prey (because prey density is the main factor that influences the functional response) in the same environment and count the number of prey eaten. Then, various functional response models are fit to the data (Okuyama & Ruyle, 2011). When the functional response *f* is independent of the predator density *P* (e.g., Holling, 1959), studies typically do not consider testing the assumption that *fP* describes the predation rate when there is more than one predator (*P *>* *1), after characterizing *f* when *P *=* *1 (i.e., one predator in an experimental arena). Nevertheless, regardless of how well a model might describe the predation rate when there is one predator, there is no guarantee that the predation rate can be extrapolated by *fP* when there are multiple predators. Such neglect of model testing suggests that there might be little interest in the validity of the per capita assumption.

The per capita emphasis is the same when predator‐dependent models (e.g., Arditi & Akçakaya, 1990; Arditi & Ginzburg, 1989; Beddington, 1975; DeAngelis, Goldstein, & O'Neil, 1975) are considered. In studies that consider predator‐dependent functional responses, multiple levels of predator densities are tested by assuming that *fP* (in which *f* is a predator‐dependent functional response) describes the predation rate of a predator population (e.g., Elliott, 2003; Hossie & Murray, 2016), but potential variation among predators is neglected. For example, when nine prey are eaten by three predators in a trial, one predator eating nine prey with the rest eating no prey and each predator eating three prey result in identical data. The per capita‐based models (e.g., *fP*) assume that such variation is not important and that the average foraging success *f* can accurately describe the process. In fact, it can be argued that variation among predators is not important as long as a model can accurately predict the number of prey eaten (e.g., for prey population dynamics, which predator ate which prey is not important). However, that argument does not hold when we start considering the numerical response of predators.

The numerical response β is usually modeled as a function of functional response because reproduction requires the energy obtained by foraging (Hessen, 1992; Humphreys, 1979). It is most commonly assumed that β = *bf*, where *b* is the conversion efficiency (the ability of predators to convert consumed prey to offspring) (e.g., Case, 2000; McCann, 2012; Murdoch et al., 2003). However, β and *f* can be related nonlinearly (e.g., Crawley, 1975). For example, parasitoid wasps are often egg limited (Heimpel & Rosenheim, 1998), setting a constraint on reproduction. Time (e.g., required to develop or lay eggs) can also be a constraint (Kokwaro, 1983). Reproductive limitations imply that the relationship between β and *f* will not be indefinitely linear and will likely be concave, at least when the foraging success is sufficiently high.

The relationship between the numerical response β and the functional response *f* can have important effects when we consider variation in the foraging success among predators (Okuyama, 2013). The effects emerge through Jensen's inequality and can be illustrated as follows. Suppose that predation success is variable among predators and that *f*_*i*_ describes the foraging success of the *i*th predator. Then, the total reproductive output of the predator population is $\sum_{i=1}^{P}\beta \left(f_{i}\right)$. On the other hand, per capita‐based modeling will predict β(*f*)*P*, where *f* is the average of *f*_*i*_. These two quantities are the same only when *f*_*i*_ is the same for all *i* (no individual variation) or when the relationship between β and foraging success is linear. However, as discussed above, both conditions are almost inevitably violated. Despite its potential importance, there is little information on variation in foraging success among predators.

This study quantifies variation in the foraging success among predators in a setting that is commonly used in laboratory studies of functional response. When *n* prey are consumed by *P* predators, *n*/*P* prey are consumed on average by each predator, no matter which functional model is considered. Because the concept is invariant to functional response models, the study focuses solely on individual variation without considering functional response. A simple model describing variation in the number of prey captured among predators is a multinomial model, in which each consumed prey is randomly allocated to predators (the model is discussed in detail below). Whether observed variation in the foraging success among predators is greater than the variation expected by the multinomial model and whether the variation changes with predator density were examined. A population model incorporating variable predators is developed and explored to examine the effects of the variation on population dynamics.

## 2 Materials and Methods

### 2.1 Predation experiment

To quantify the level of individual variation in foraging success among predators, the predation of the wolf spider *Pardosa pseudoannulata* on the cricket *Gryllus bimaculatus* was examined. The aim of the study was to examine the level of individual variation, rather than quantifying the average predation rate (i.e., functional response). Therefore, the average predation rate was fixed in the experiment. In a trial, *P* predators and *N* prey (specific values for *P* and *N* are described below) were introduced in an experimental arena (10 cm × 14 cm; height = 7.8 cm), and predation events were monitored using a video camera such that it was possible to quantify the number of prey consumed by each predator. All experiments began between 09:00 and 10:00 hr and ended within 9 hr in a temperature controlled (≈26°C) room. Three levels of predator density *P *=* *2,3, and 4 were tested to examine the effect of predator density on foraging success variation. To control the average foraging success, the numbers of prey were fixed at *N *=* *3*P* such that one spider ate three prey on average (in all trials, all prey were eaten). The number of replications for the three predator levels *P *=* *2, 3, and 4 were 25, 25, and 23, respectively. Each spider was used only once in the study. The experimental design and structure (e.g., arena size) described above is comparable to those of conventional functional response studies (e.g., Cave & Gaylor, 1989; Jalali, Tirry, & De Clearcq, 2010; Khan, 2013; Líznarová & Pekár, 2013). This makes it possible to quantify the level of foraging success variation that might be common in numerous existing functional response studies in which the variation is not recorded (e.g., for logistical reasons).

Variation in the foraging success among spiders was intentionally minimized by the experimental design. First, all spiders were similarly sized immature individuals (average carapace width 1.8 mm, *SD* = 0.2 mm). Small spiders were used to minimize the effect of the small arenas. Using similarly sized individuals also eliminated the occurrence of cannibalism (none was observed in all trials). Prior to the trial, all spiders were starved for 7 days after they were satiated to control their satiation level. All prey crickets were the first instar. The minimized variation allowed us to quantify the minimal level of foraging variation. For example, for a given level of variation characterized by the experiment, we would expect at least the same (but likely a much higher) level of variation to be realized in the field.

### 2.2 Statistical analysis

Based on the experimental design, the response variable is a vector of the numbers of prey eaten by each predator **y** = (*y*_1_,…, *y*_*P*_), where *y*_*i*_ is the number of prey consumed by the *i*th predator and $\sum_{i}y_{i}=3P$. When each of 3*P* prey is randomly allocated to a predator, the values follow a multinomial distribution. Under the multinomial model, the likelihood can be calculated using a probability mass function (shown below), whose size parameter is 3*P*, and the probability vector **p **= (*p*_1_,…, *p*_*P*_) is a *P*‐tuple of 1/*P* (i.e., *p*_*i*_ = 1/*P* for all *i*). Thus, the model has no free parameter to be estimated.

To examine whether the observed variation is greater than that expected on the basis of the multinomial model, the Dirichlet‐multinomial distribution was also examined (Johnson, Kotz, & Balakrishnan, 1997). The Dirichlet‐multinomial distribution is a compound distribution in which the probability vector of the multinomial distribution is not fixed but follows another probability distribution, a Dirichlet distribution. The parameters of a Dirichlet distribution are a vector **α **= (α_1_, α_2_, …, α_*P*_). In this study, α_*i*_ = α for all *i* is assumed, indicating that the expected number of prey captured by the each predator is the same.

Specifically, the probability mass functions of the multinomial distribution *f*_M_ and the Dirichlet‐multinomial distribution *f*_DM_ are

(1) $$f_{M}\left(y_{1},\ldots ,y_{P}|N,p\right)=\frac{N!}{y_{1}!\ldots y_{P}!}\prod_{i=1}^{P}\frac{1}{P^{y_{i}}}$$

(2) $$f_{\mathrm{DM}}\left(y_{1},\ldots ,y_{P}|N,\alpha \right)=\frac{\left(N!)\Gamma (\alpha P\right)}{\Gamma (N+\alpha P)}\prod_{i=1}^{P}\frac{\Gamma (y_{i}+\alpha )}{\left(y_{i}!\right)\Gamma \left(\alpha \right)}$$

where *N *=* *3*P* in this study. In both cases, the expected number of prey for each predator is *E*_M_(*Y*_*i*_) = *E*_DM_(*Y*_*i*_) = *N*/*P *=* *3 for all *i*. The variances for the multinomial and the Dirichlet‐multinomial models, respectively, are

(3) $$V_{M}\left(Y_{i}\right)=N\frac{1}{P}\left(1-\frac{1}{P}\right)$$

(4) $$V_{\mathrm{DM}}\left(Y_{i}\right)=N\frac{1}{P}\left(1-\frac{1}{P}\right)\left(\frac{N+\alpha P}{1+\alpha P}\right)$$

for all *i*. The variance of the Dirichlet‐multinomial distribution is always greater than that of the multinomial distribution (i.e., (*N* + α*P*)/(1 + α*P*) > 1). When α = ∞, the Dirichlet‐multinomial model converges to a multinomial distribution. The two models were compared using the likelihood ratio test. Rejection of the multinomial model indicates that the observed variation in the foraging success among predators is greater than would be expected by the random allocation model of the multinomial process.

### 2.3 The population model

A model consisting of one predator species and one prey species was used to examine the effect of variation in foraging success among predators. The dynamics of populations are described by

(5) N(t+1)=R(t)

(6) P(t+1)=B(t)+[P(t)−D(t)]

where the density of prey and predator at time *t* are *N*(*t*) and *P*(*t*), respectively. *R*(*t*), *B*(*t*), and *D*(*t*) are random variables that describe the recruitment of the prey, the number of predator offspring, and the number of predator deaths, respectively. In each time step, predation, reproduction, and predator death take place in this order. The predator is assumed to die in a density‐independent manner. *D*(*t*) follows a binomial distribution whose size parameter is *P*(*t*), and the probability parameter is *e* ^___*m*^, where *m* is the per capita mortality rate. The prey population grows in a self‐limiting manner, for which the Beverton___Holt model was used (Beverton & Holt, 1957). The expected size of the prey population in the next time step is

(7) $$E\left(R\left(t\right)\right)=\left(\frac{e^{r}S\left(t\right)}{1+\left[\left(e^{r}-1\right)/K\right]S\left(t\right)}\right),$$

where *r* and *K* are the intrinsic rate of increase and the carrying capacity of the prey, respectively. *S*(*t*) is the number of prey that survive predation in the current time step (described below). *N*(*t *+* *1) is simulated from a Poisson distribution with the specified expectation, *E*(*R*(*t*)).

The expected value of the number of prey eaten by predators *Y*(*t*) is determined by a type II functional response,

(8) $$E\left(Y\left(t\right)\right)=N\left(t\right)-\frac{1}{ah}\text{Lambert}W\left(ahN\left(t\right)e^{-a(P(t)-hN(t))}\right),$$

where *a* and *h* are the attack rate and the handling time, respectively, of the predator. Although the Lambert *W* function is used for convenience (Bolker, 2008; Okuyama & Ruyle, 2011), the same solution can be obtained using the random predator equation (Rogers, 1972). The actual number of prey eaten is simulated from a binomial distribution whose size parameter is *N*(*t*), and the probability parameter is *E*(*Y*(*t*))/*N*(*t*). Supposing that *y*(*t*) is a realization of the random variable *Y*(*t*) (i.e., the total number of prey eaten by the predator population), the number of surviving prey used in equation (8) can be calculated as *S*(*t*) = *N*(*t*) − *y*(*t*).

If we assume that each predator receives an equal amount of resources, then *y*(*t*)/*P*(*t*) is consumed by each predator. The aim of the population model is to relax this assumption and allow for variability among predators. When multinomial variation is assumed,

(9) $$\left(Y_{1}\left(t\right),Y_{2}\left(t\right),\ldots ,Y_{P(t)}\left(t\right)\right)∼\text{Multinominal}(y(t),p),$$

where *Y*_*i*_(*t*) is the random variable describing the number of prey captured by the *i*th predator, *p*_*i*_ = 1/*P* for all *i*, and $\sum_{i}Y_{i}\left(t\right)=y\left(t\right)$. *p*_*i*_ is the probability that a prey is captured by the *i*th predator, and thus the model describes the random distribution of the prey among the predators. The Dirichlet‐multinomial distribution can also be used instead of a multinomial distribution.

(10) $$\left(Y_{1}\left(t\right),Y_{2}\left(t\right),\ldots ,Y_{P(t)}\left(t\right)\right)∼\text{Dirichle‐multinominal}(y(t),\alpha ),$$

where α_*i*_ = α for all *i*. As discussed above, the two models are the same when α = ∞.

The relationship between reproduction and foraging success for a predator is assumed to be a concave function, *by*_*i*_(*t*)/(*q* + *y*_*i*_(*t*)), where *b* and *q* are the parameters that shape the relationship, and *y*_*i*_(*t*) is the number of prey eaten by the *i*th predator individual (i.e., *y*_*i*_(*t*) is a realization of the random variable *Y*_*i*_(*t*)). The number of offspring, *B*_*i*_(*t*), produced by the *i*th predator is a random variable that follows a Poisson distribution with mean *by*_*i*_(*t*)/(*q* + *y*_*i*_(*t*)). Therefore, the total number of offspring is

(11) $$B\left(t\right)=\sum_{i=1}^{P(t)}B_{i}\left(t\right),$$

which completes the model specification.

The population model was used to understand the effect of individual variation on population dynamics through the numerical response *B*(*t*). Therefore, the dynamics under three different models were compared: (1) no variation (*Y*_*i*_ = *y*_*i*_(*t*)/*P*(*t*) for all *i*), (2) intermediate variation (*Y*_*i*_ follows a multinomial distribution), and (3) strong variation (*Y*_*i*_ follows the Dirichlet‐multinomial distribution). The responses of the three models to environmental enrichment (i.e., increase in *K*) were compared.

## 3 Results

### 3.1 Predation experiment

**Figure 1:**
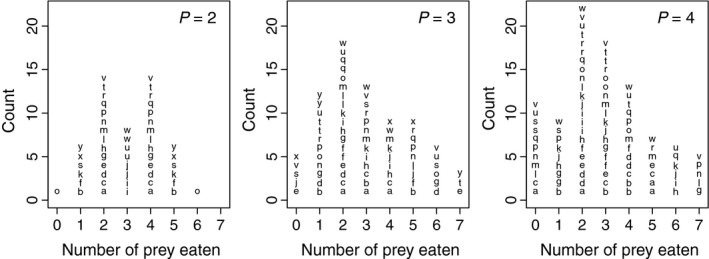
Raw data in dot plots for the three levels of predator density (*P *=* *2, 3, and 4). The same letter (within the same *P*) indicates the same replication. For example, in *P *=* *3, the letter “a” is seen for two, three, and four (number of prey eaten), indicating that the three spiders in the group ate these numbers of prey, respectively. When *P *=* *4, one spider ate 11 prey (group “s”) that are outside the plotted range

The raw data of the experiment are shown in Figure 1. When the predator density was high (*P *=* *3 and 4), the Dirichlet‐multinomial model better described the data than the multinomial model (Table 1; likelihood ratio test, *p *<* *.05). When *P *=* *4, one individual ate 11 prey, which might be considered an outlier, but removing this sample does not change the conclusion of the analysis. When *P *=* *2, the multinomial model was not rejected.

**Table 1:** Summary of statistical tests. *P*: number of predators. *L*_M_: negative log‐likelihood for the multinomial model. *L*_DM_: negative log‐likelihood for the Dirichlet‐multinomial model. α: maximum‐likelihood estimate for the Dirichlet‐multinomial model. *D*: test statistic for the likelihood ratio test. When *p*‐value is <.05, the multinomial model is rejected

*P*	*L*_M_	*L*_DM_	α	*D*	*p*‐value
2	43.33	42.9	9.48	0.85	.85
3	99.65	94.54	2.43	10.24	.001
4	144.84	135.14	2.81	19.40	1.06 × 10^−5^

As predator density increased, the parameter, α, of the Dirichlet‐multinomial model decreased (Table 1), indicating that the variation in the foraging success is density‐dependent and becomes stronger as the predator density increases. However, even when α is constant over predator density, variation in the foraging success is still density‐dependent (discussed further below).

### 3.2 Population dynamics

Increasing the carrying capacity *K* destabilizes predator–prey dynamics, a well‐known result (Rosenzweig, 1971). How variation in foraging success might influence the effect of enrichment was examined.

**Figure 2:**
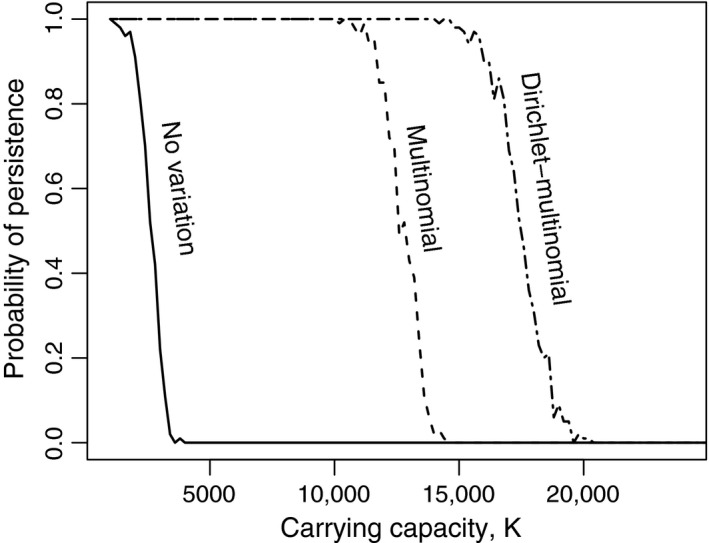
Effects of carrying capacity on persistence possibility. Successful persistence indicates the persistence of the two species for 10,000 time steps. The probability of persistence was calculated based on 100 independent simulation runs. For the Dirichlet‐multinomial model, α = 1 is used. Parameters: *a *=* *0.001, *h *=* *0.05, *b *=* *0.15, *r *=* *.5, *m *=* *0.05, *q *=* *0.25

Variation among predators makes community persistence (i.e., both the predator and prey persist without becoming extinct) robust to environmental enrichment. Although persistence becomes impossible when *K* is sufficiently high, regardless of the level of predator variation, the possibility of persistence is extended to much higher levels of *K* when variation in foraging success among predators is high (Figure 2).

**Figure 3:**
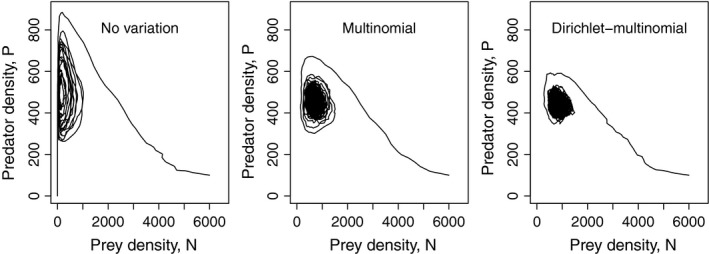
Typical dynamics under three different models of foraging success. In each simulation, the initial density was *N *=* *6,000 and *P *=* *100. When there is no variation (left figure), the population eventually goes to extinction (i.e., *P *=* *0 and *N *=* *0). For the Dirichlet‐multinomial model, α = 1 is used. Simulations were run for 5,000 time steps. Parameters: *a *=* *0.001, *h *=* *0.05, *b *=* *0.15, *r *=* *.5, *m *=* *0.05, *q *=* *0.25, *K *=* *5,000

When persistence is possible, the dynamics are cyclic, partly owing to demographic stochasticity (McKane & Newman, 2005; Okuyama, 2015a). As variation in the foraging success among predators increases, the amplitude of nonequilibrium dynamics decreases, and the minimum prey population densities become high (e.g., random prey extinction becomes less likely) (Figure 3).

**Figure 4:**
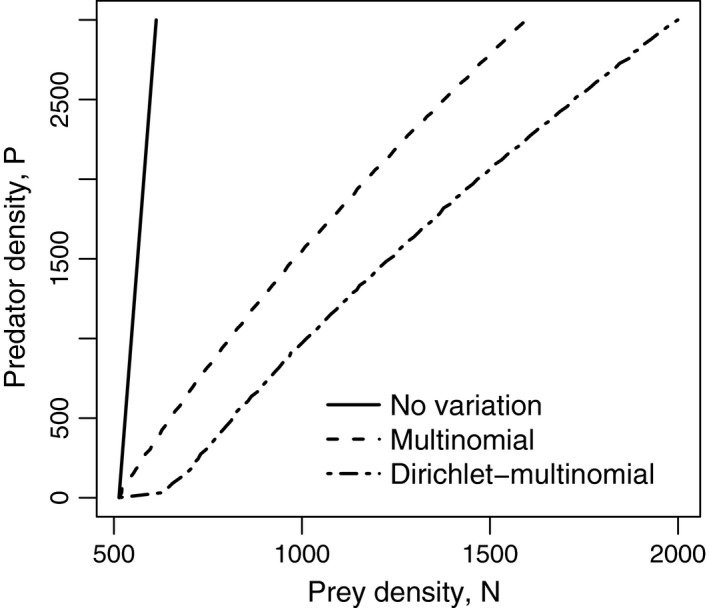
Predator isoclines for three models. When the combination of prey density *N* and predator density *P* is on the right side of the line, the predator density is expected to increase. On the left side, the density will decrease. Parameters: *a *=* *0.001, *h *=* *0.05, *b *=* *0.15, *m *=* *0.05, *q *=* *0.25

The effect of variability among predators on stability is revealed by predator isoclines (conditions that satisfy a zero growth rate, *P*(*t *+* *1)/*P*(*t*) = 1). The population growth rate of the predator *P*(*t *+* *1)/*P*(*t*) was numerically estimated for various combinations of *P*(*t*) and *N*(*t*). Before discussing the effect of variable predators, it is important to note that the model contains predator–predator interactions even without variation among predators (i.e., the tilted isocline in Figure 4). This density dependence will not appear in the continuous model. If we use a continuous model,

(12) $$\frac{dP}{dt}=\beta \left(f\right)P-mP,$$

whether β is linear (e.g., β(*f*) = *bf*) or nonlinear (e.g., β(*f*) = *bf*/(*q* + *f*)), the predator isoclines (*dP*/*dt* = 0) are vertical, and predator density does not influence the sign of *dP*/*dt*, provided that there is no individual variation among predators and that *f* is independent of predators (e.g., a type II functional response model). Thus, the way the model was formulated (i.e., prey are depleted within a time step; equation (9)) introduced a density dependence that would otherwise not exist.

The mechanism in which variation in foraging success enhances persistence is that the variation will strengthen the predator–predator interaction. This is seen in the effect of the variation on the slope of the isocline (Figure 4). As the variation becomes stronger, the isocline is more strongly tilted. This negative density dependence (i.e., self‐limitation) is a general stability mechanism for many consumer‐resource dynamics (Case, 2000; Hastings, 1997).

## 4 Discussion

Variation in the number of prey consumed by predators is generally assumed to be unimportant in population dynamics. Consequently, we have little information regarding variability among predators. This study revealed the presence of a large variation in foraging success among predators. In particular, for a given number of prey consumed by a group of spiders, variation in the number of prey eaten by each predator is greater than that expected on the basis of random assignment of prey to predators. A mathematical model that incorporates predation variability indicates that this variation can stabilize predator–prey interactions. These results suggest that understanding factors that create variability in foraging success are important for developing a mechanistic understanding of population and community dynamics.

In the laboratory experiment, various factors were standardized to minimize variation in results, but significant variation (e.g., multinomial and greater) was observed. One could argue that at least the multinomial variation is expected naturally. However, this is not the case (more significantly, it being the case would further strengthen the importance). Under the conventional predation scheme in which the functional response was developed, a predator is either searching for prey or handling prey (Holling, 1959; Okuyama, 2012). Supposing that there are three predators, and only one of them is currently handling a prey, then the next prey is most likely consumed by one of the searching predators. That is, allocation of prey is not random but systematic. This systematic expectation holds for most conventional functional response models, including predator‐dependent ones. Therefore, models predict more uniform foraging success among predators than that expected on the basis of the multinomial model. Furthermore, high variability was observed despite the fact that variability in the amount of time required to find a prey is minimized by the use of the simple experimental arena. These results suggest that the predation process is more complex than the common assumptions used to develop functional response models suggest.

The study considers all predators are identical, and the variation in foraging success results by chance. However, another factor that contributes to the observed variation is the actual difference among individuals. For example, some individuals might have been more aggressive than others (e.g., Mazué, Dechaume‐Moncharmont, & Godin, 2015; Pellegrini, Wisenden, & Sorensen, 2010), creating uneven predation success among individuals. Although functional response is not considered in the study (i.e., the average number of prey eaten was fixed), it is likely that predator–predator interactions (e.g., Arditi & Akçakaya, 1990; Arditi & Ginzburg, 1989; Beddington, 1975; DeAngelis et al., 1975) influenced the observed variability in predation. Although the analysis assumed *p*_*i*_ = 1/*P* for all *i*, if there is a true difference between individuals (e.g., *p*_1_ > *p*_2_ when *P *=* *2), it will further increase the forging variation among predators.

The mathematical model shows that individual variation will stabilize predator–prey interactions (Figure 2). One factor is Jensen's inequality (Ruel & Ayres, 1999). Because the relationship between numerical and functional response is assumed to be concave as a result of some reproductive limitation, the presence of individual variation in functional response will decrease the reproduction rate of the predator population (Okuyama, 2013). Another important factor is the multinomial (or the Dirichlet‐multinomial) model used to describe variability among predators (Okuyama, 2015b). In the population model, it was assumed that the functional response model can predict the number of prey consumed by the population of predators accurately (equation (9)). Given a number of prey consumed, the multinomial‐type models are convenient for describing how the prey are allocated to existing predators. One of the characteristics of these models is that as the number of elements (i.e., *P* in the model) increases, variation among the elements of a response also increases (for a fixed mean). For example, when the total number of prey eaten is *y* and each predator eats *y*_*i*_ prey (*i *=* *1,…,*P*) such that $\sum_{i}y_{i}=y$, the variation among predators in the number of prey eaten (i.e., the variability of y_1_, …,y_*p*_) increases as *P* increases (when *p*_*i*_ = 1/*P* for all *i*). The same is true for the symmetric Dirichlet‐multinomial model for a constant, α_*i*_ = α for all *i*. In other words, variability in foraging success among predators increases with predator density. This is why even when α is constant for levels of *P*, this does not indicate that variation is density‐independent, and the density‐dependent α characterized by the experiment (Table 1) strengthens the pattern further.

Spiders are known to exhibit partial consumption (e.g., Pollard, 1988; Samu, 1993) in which prey are only partially consumed. Therefore, we cannot simply assume a linear relationship between the number of prey eaten and the energy gain as assumed in the population model. The effect of partial consumption is likely complex. It may decrease the variation in energy gain as successful foragers may not consume much energy from each prey. At the same time, partial consumption may increase the variation in energy gain if partially consumed prey become unavailable to other predators. Although the occurrence of partial consumption certainly makes the relationship between foraging success and energy gain more complex, the concept discussed in this study is still valid. Whether the presence or absence of partial consumption, it is still unreasonable to assume that the energy gain is identical among all predators. In addition, although spiders were used as the study subject, the stability mechanism discussed here applies to any consumers including ones that do not exhibit partial consumption.

Despite the recognition of the importance of individual variation, quantifying foraging variability among predators is difficult. In this study, accurate quantification was possible because the environmental arena was sufficiently simple that the entire space was clearly monitored by video recording. In the field, tracking the same individual over time and recording its foraging events are difficult to impossible, despite advancements in the technology for tracking the positions of individuals (Cooke et al., 2004, 2013). One possible resolution is that if it is possible to record the same individual over time, recording the body weight of an individual over time will give some information about its foraging success (Jakob, Marshall, & Uetz, 1996), thereby allowing estimation of individual variability. Another approach for examining the prediction/assumption of the study is to test the per capita assumption discussed in *Introduction*. Even when it is impossible to quantify individual‐level data, it is often possible to quantify population‐level responses (e.g., common functional response studies). This allows testing of whether β*P* can accurately predict the numerical responses for various levels of the predator density *P*.

Individual variation is recognized as an important factor in ecological processes (Bolnick et al., 2011), but we still largely lack concrete theory to connect variation with explicit ecological predictions. Consequently, even though many empirical studies have begun quantifying individual variation, its roles are still unclear despite acknowledgment of its importance. This study described how individual variation can create density‐dependent interactions among predators and showed that a simple fundamental reality (i.e., foraging variation among predators) has a profound effect on population dynamics. Generating testable predictions and refining the models of individual variation can be expected to be a promising approach for the development of a mechanistic theory of population dynamics.

## Conflict of Interest

None declared.
